# Early Fault Detection Method for Rotating Machinery Based on Harmonic-Assisted Multivariate Empirical Mode Decomposition and Transfer Entropy

**DOI:** 10.3390/e20110873

**Published:** 2018-11-13

**Authors:** Zhe Wu, Qiang Zhang, Lixin Wang, Lifeng Cheng, Jingbo Zhou

**Affiliations:** 1 School of Mechanical Engineering, Hebei University of Science and Technology, Shijiazhuang 050018, China; 2 School of Mechanical and Vehicle Engineering, Beijing Institute of Technology, Beijing 100081, China; 3 Key Laboratory of Vehicle Transmission, China North Vehicle Research Institute, Beijing 100072, China

**Keywords:** HA-MEMD, rotating machinery, transfer entropy, fault diagnosis

## Abstract

It is a difficult task to analyze the coupling characteristics of rotating machinery fault signals under the influence of complex and nonlinear interference signals. This difficulty is due to the strong noise background of rotating machinery fault feature extraction and weaknesses, such as modal mixing problems, in the existing Ensemble Empirical Mode Decomposition (EEMD) time–frequency analysis methods. To quantitatively study the nonlinear synchronous coupling characteristics and information transfer characteristics of rotating machinery fault signals between different frequency scales under the influence of complex and nonlinear interference signals, a new nonlinear signal processing method—the harmonic assisted multivariate empirical mode decomposition method (HA-MEMD)—is proposed in this paper. By adding additional high-frequency harmonic-assisted channels and reducing them, the decomposing precision of the Intrinsic Mode Function (IMF) can be effectively improved, and the phenomenon of mode aliasing can be mitigated. Analysis results of the simulated signals prove the effectiveness of this method. By combining HA-MEMD with the transfer entropy algorithm and introducing signal processing of the rotating machinery, a fault detection method of rotating machinery based on high-frequency harmonic-assisted multivariate empirical mode decomposition-transfer entropy (HA-MEMD-TE) was established. The main features of the mechanical transmission system were extracted by the high-frequency harmonic-assisted multivariate empirical mode decomposition method, and the signal, after noise reduction, was used for the transfer entropy calculation. The evaluation index of the rotating machinery state based on HA-MEMD-TE was established to quantitatively describe the degree of nonlinear coupling between signals to effectively evaluate and diagnose the operating state of the mechanical system. By adding noise to different signal-to-noise ratios, the fault detection ability of HA-MEMD-TE method in the background of strong noise is investigated, which proves that the method has strong reliability and robustness. In this paper, transfer entropy is applied to the fault diagnosis field of rotating machinery, which provides a new effective method for early fault diagnosis and performance degradation-state recognition of rotating machinery, and leads to relevant research conclusions.

## 1. Introduction

Components like rolling bearings and gears are the most extensively used and vulnerable components in a mechanical transmission system, and they may frequently fail under highly variable loads resulting from complex operating conditions. Vibration analysis technology has been extensively used for mechanical fault diagnosis and mode recognition [1,2]. The existence of various complex excitations and strong noise in vibration signals tremendously limit the possibility of extracting effective rotating machinery fault features from the original vibration signal, impairing the accuracy rate of fault diagnosis [3]. Hence, it is necessary to use nonlinear research methods to identify bearing and gear faults so as to improve the signal-to-noise ratio of vibration signals. As the equipment becomes increasingly complex, it becomes progressively more difficult to extract the features accurately. How to extract fault feature information of a bearing has effectively become the core problem of bearing fault diagnosis [4].

In recent years, fault diagnosis technology for rotating machinery has developed rapidly. Researchers have conducted many investigations on the condition assessment and diagnosis of rotating machinery faults based on vibration signals [5,6]. Singh [7] combined continuous wavelet transform and angle resampling for gearbox fault diagnosis under variable working conditions. However, wavelet transform requires the setup of a wavelet base and parameters, since it is not capable of adaptive data processing. Empirical Mode Decomposition (EMD) [8] and Local Mean Decomposition (LMD) [9] are data-driven adaptive nonlinear analysis methods proposed respectively by Huang and Smith in 1998 and 2005. EMD adaptively decomposes a nonstationary nonlinear signal into a series of Intrinsic Mode Function components with clear instantaneous frequency and physical significance. Guo [10] proposed an improved empirical mode decomposition method based on multi-objective optimization. Particle Swarm Optimization (PSO) was used to find the optimal intrinsic mode function (IMF) and determine the optimal shape control parameters. The vibration signal of the failed rolling bearing is effectively extracted. Despite the fact that EMD and LMD have a good many merits, EMD exhibits defects, such as mode mixing and end effect, so as to affect the noise reduction effect and fault state identification of the vibration signal of the mechanical transmission system under complex operating conditions.

To avoid mode mixing when EMD is used in an environment where a decomposition intermittent noise signal exists, Huang and Wu proposed the Ensemble Empirical Mode Decomposition (EEMD) method in 2009: by adding white noise to the target signal, EEMD enables a uniform ratio of signal in time–frequency space. As a noise-assisted data analysis technique, EEMD was developed to successfully suppress mode mixing [11,12,13]. Amarnath [14] used EEMD to extract fault-related characteristics from the vibration signal acquired from a gearbox; furthermore, this research took into account the estimation of the specific lubricating film thickness and the effect on the increase of the gear tooth surface fault. Support Vector Machine (SVM) [15] is a machine-learning algorithm proposed by Vapnik based on the principle of structural risk minimization. Tabrizi [16] denoised the early fault signal of bearings using EEMD and combined it with an SVM to propose an automatic detection method of bearing microdefects based on EEMD and SVM.

To further improve the decomposition capacity of LMD and achieve decomposition results with more accurate physical significance, Sun [17] added white noise with a limited amplitude to the target signal and averaged the results of several decompositions, thereby proposing Ensemble Local Mean Decomposition (ELMD). Soon after, ELMD, as an improvement of LMD, was successfully introduced to the field of fault diagnosis and state monitoring for rotating machinery [18]. The ELMD method is largely dependent on the correct selection of the model parameters. Zhang et al. [19] used the error index of relative Root Mean Square Error (RMSE) and the Signal-to-Noise Ratio (SNR) as indicators for signal decomposition effect evaluation, and, having identified an optimal set of ELMD parameters, proposed an optimized integrated LMD technique.

By integration averaging, EEMD and ELMD eliminated the white noise components contained in IMF, which, to some extent, reduced the mode mixing phenomenon, but integration averaging led to the generation of a new endpoint effect and a longer operation time [20]. Rehman [21] proposed the Multivariate Empirical Mode Decomposition (MEMD) algorithm to make up for the drawbacks of EMD, i.e., that it can only process one-dimensional signals. MEMD enables multichannel simultaneous signal analysis. As an extension of multichannel signal processing, MEMD offers an effective means of gaining insight into complicated nonstationary nonlinear real signals [22,23]. Research scholars have successfully applied the MEMD method to EEG signal [24] and mechanical signal processing [25]. As a new time–frequency analysis algorithm, MEMD encounters some technical barriers in practical situations, such as modal aliasing, which hinders its development in the signal processing field and impedes further application to mechanical fault diagnosis.

To mitigate the modal mixing of MEMD, Rehman [26] proposed Noise-assisted Multivariate Empirical Mode Decomposition (NA-MEMD), which added several auxiliary white noise channels to the decomposition and achieved the oscillating mode multivariate of the corresponding IMFs from several signal channels through averaging. As opposed to EEMD, the white noise is not directly added to the target signal; hence, NA-MEMD is not encumbered by the various problems of EEMD mentioned above. NA-MEMD is considered an effective data analysis technique, since it alleviates the spectrum loss that occurs during the decomposition of EEMD, thereby leading to a more accurate IMF spectral distribution of the decomposition result than that achieved with EEMD. Relevant studies indicate, however, that although NA-MEMD improves the modal aliasing in MEMD, it has not completely resolved this problem, so further studies are needed. Wu [27] proposed the adaptive high-frequency harmonic LMD method by adding high-frequency harmonics to the target signal and suppressing modal aliasing through the change in extreme point locations of the target signal [28].

Although accurate state recognition and feature extraction are the basis for rotating machinery fault diagnosis, it is impossible to obtain accurate state feature information merely from time-domain and frequency-domain feature information, since a rotating machinery fault signal is highly nonlinear, non-Gaussian, and nonstationary. The nonlinear characteristics of a vibration signal change with the evolution of rolling bearing and gear failures, so the most important task is to establish accurate and sensitive state regression indicators [29].

Richman [30] proposed sample entropy, a new method for measuring the complexity of a time series. Ni [31] uses Sample Entropy characteristics to detect and evaluate early faults of rolling bearings, and verifies the effectiveness of the method through wind power measured data. A new method called “permutation entropy” (PE) [32] has recently been proposed as a measure for complex nonlinear and linear time series. Tiwari [33] proposed a permutation entropy and adaptive neural fuzzy classifier (ANFC) -based bearing fault diagnosis method that employs permutation entropy for feature extraction. To alleviate the complexity of the feature vector, the extracted features are entered in to the ANFC for automatic fault diagnosis.

None of the above-noted nonlinear time series measuring methods can effectively describe the correlation between related time series. Schreiber [34] proposed a new method for measuring nonlinear system correlation in 2000, namely, Transfer Entropy, which can not only quantify the information coupling strength between the two systems, but also calculate the direction of information transfer. Since it was proposed, the Transfer Entropy has been widely used. In addition to their extensive application in the field of physical communication, the transfer entropy and related entropy theories have been employed in many fields, including chemistry, biology, and medicine [35,36,37].

As a measure of information flow between Markov processes, transfer entropy has been confirmed by recent research to be applicable to analyses of the degree of coupling in structural dynamics. Kaiser and Schreiber [38] proved that transfer entropy more appropriately quantified the dynamic relationship between time series data than mutual information; additionally, transfer entropy can capture the asymmetry of the information sharing method between two different dynamic processes. Since transfer entropy captures the dynamic dependence in a more favorable manner, it may be more suitable for determining the degree of nonlinear correlation in system dynamics. Simulation and test results show that transfer entropy is more sensitive to nonlinearity than the delayed mutual information function, thus being an effective structural damage testing tool. Nichols [39] performed an analysis based on the vibration response of a transfer entropy composite material, detected structural damage based on the change in degree of nonlinear coupling between various locations on the structure, and proved the effectiveness of the method through a spring-mass-damping model test and the impact damage test of a Unmanned Aerial Vehicle wing under an environmental gust load. It was found that transfer entropy could favorably identify the nonlinear behavior of the structure under the effect of certain noise, which demonstrated the high sensitivity and robustness of transfer entropy in structural damage identification. Liu and Xie [40] identified the damage of a concrete simple-supported beam using the mean transfer entropy at different time scales, studied the damage quantification and localization capacity of the transfer entropy, and verified the rationality of the direct use of the linearized transfer entropy theory by the kernel density estimation technique. Sun [41] proposed a transmission path identification method based on delayed transfer entropy for the vibration transmission path and the transmission direction of the coupled power system of a power generator set; the effectiveness of this method was demonstrated through simulation analysis and testing. Although transfer entropy has been successfully used in the field of structural damage testing, the nonparametric one-, two-, and three-dimensional probability density functions must be estimated for the calculation of transfer entropy; hence, external noise-induced random disturbances may result in significant differences in transfer entropy. Overbey evaluated the effect of input and output noise on transfer entropy through numerical simulation of a spring oscillator system and steel frame test, and studied the damage identification capacity of transfer entropy in the presence of noise by adding noises with different SNRs; it was found that the sensitivity of the transfer entropy in the estimation of the damage characteristics was reduced by generating low variance through the reduction of [42].

With the occurrence and development of an internal fault in components such as bearings and gears in rotating machinery in the running state, a strong nonlinear relationship exists between its vibration signal characteristics and the running state, so a nonlinear research approach must be used to identify a gear fault. Most recognition methods for existing rotating machinery fault diagnosis are not capable of simultaneous fault quantification and location, so the introduction of transfer entropy theory into the field of rotating machinery fault diagnosis is of great theoretical and practical significance.

To quantitatively study the nonlinear synchronous coupling characteristics and information transfer characteristics of rotating machinery fault signals between different frequency scales under the influence of complex and nonlinear interference signals, a rotating machinery fault testing method based on high-frequency harmonic-assisted multivariate empirical mode decomposition-transfer entropy is proposed for the quantitative study of the nonlinear synchronous coupling characteristics and information transfer between the rotating machinery fault signal and zero fault between different time–frequency scales. The running status of the mechanical system was effectively evaluated and diagnosed through the quantitative description of the degree of nonlinear coupling between signals by (1) extracting the principal characteristics of the mechanical transmission system through high-frequency harmonic-assisted multivariate empirical mode decomposition; (2) subjecting the denoised signal to transfer entropy calculation; and (3) establishing an HA-MEMD-TE (High-frequency harmonic-assisted multivariate empirical mode decomposition-transfer entropy)-based rotating machinery status evaluation index. This study employed transfer entropy for rotating machinery fault diagnosis, providing a new effective means and relevant research findings for the early fault diagnosis and performance degradation status identification of rotating machinery.

## 2. HA-MEMD

**Figure 1:**
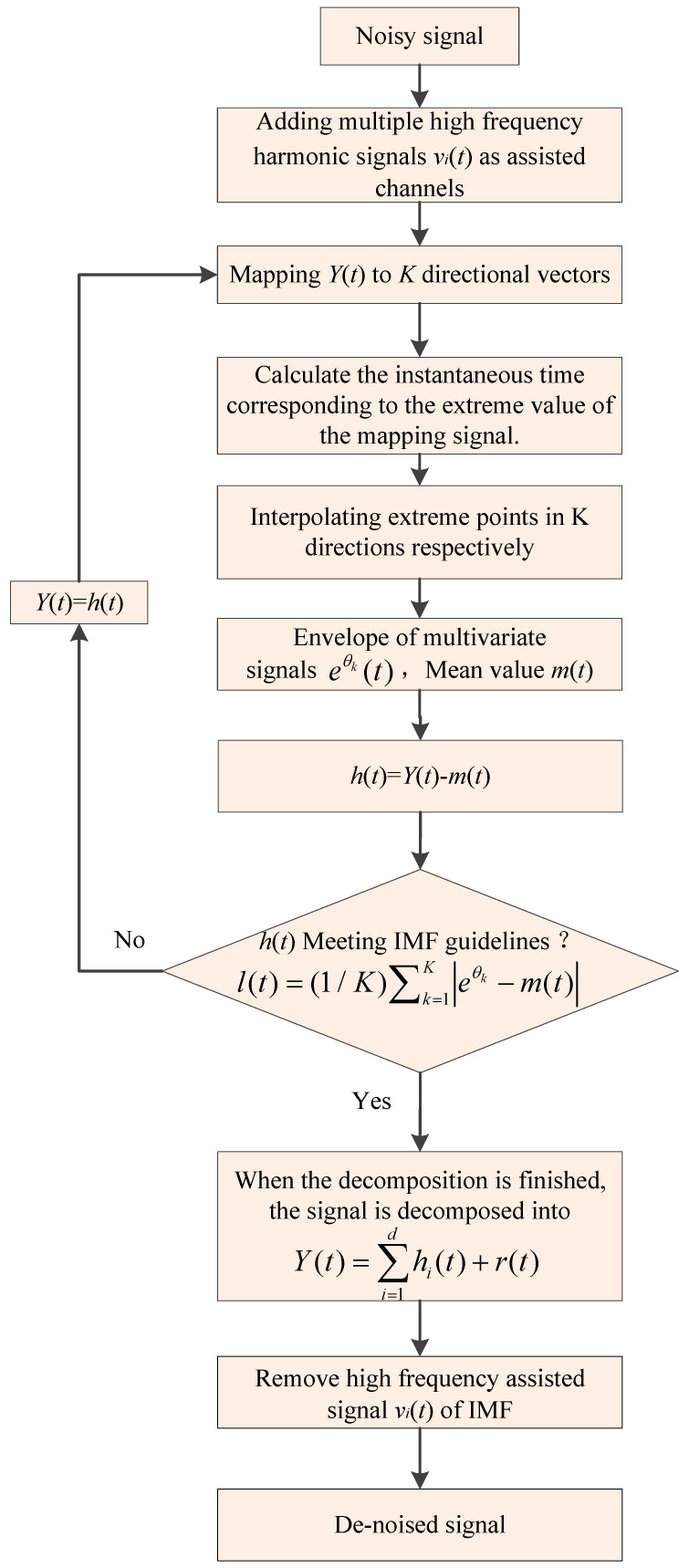
Flowchart of the HA-MEMD method.

The HA-MEMD method adds a number of high-frequency harmonic channels as auxiliary channels, establishes evenly distributed reference scales, decomposes the compound channel signal to get multichannel independent multivariate IMF components, and finally, removes the IMFs from the auxiliary channels and retains the IMFs of the target signal. The general steps of the HA-MEMD algorithm are as follows (As shown in Figure 1):

(1) Add *n* high-frequency harmonic sequences *v_i_*(*t*) (*i* = 1, 2, 3…*n*) to the target signal *X*(*t*) of length *T* to form the *n* + 1 time series *Y*(*t*).

(2) Using the hammersley importance sampling method [26] to obtain a uniform sample point set on the (*n* + 1)-dimensional spherical surface, and obtain *n* + 1 vectors.

(3) Calculate the mapping $b^{\theta_{k}}(t)$ of the input signal *Y*(*t*) on each direction vector $x^{\theta_{k}}$, where $x^{\theta_{k}}$ indicates the direction angle in the (*n* + 1)-dimensional unit ball.

(4) Find all the maximum and minimum values of the mapping signals of all directional vectors $b^{\theta_{k}}(t)$ and corresponding moments $t_{l}^{\theta_{k}}$, where *l* indicates the location of extreme points.

(5) Obtain *K* multivariate envelopes $a^{\theta_{k}}(t)$ by using the multivariate spline interpolation function value for all extreme points. The mean value of them (*t*) signal is:

(1) $$Y(t)=\frac{1}{K}\sum_{k=1}^{K}a^{\theta_{k}}(t)$$

(6) Define the IMF function *h*(*t*), which is extracted by *h*(*t*) = Y(*t*) − *m*(*t*).

(7) Defining the decomposition judgment function as f(t)=|m(t)/l(t)|, when f(t) is less than the specified threshold, the first IMF component has been successfully extracted. Subtracting the first extracted IMF *h*_1_(*t*) component from the original signal *Y*(*t*), as the input signal of step (3), continue the iterative calculation of steps (3)–(7) to extract the new multivariate IMF component *h*(*t*), l(t) is:

(2) $$l(t)=(1/K)\sum_{k=1}^{K}\left|e^{\theta_{k}}-m(t)\right|\;$$

(8) If *h*(*t*) does not satisfy the IMF criterion, use *h*(*t*) as the input of the step (3) signal, continue the (3)–(7) step iteration, then cycle *i* times until *h*(*t*) satisfies the IMF criterion.

After a series of HA-MEMD decomposition processes, the original *n* + 1 element signal is decomposed into a series of IMF $h_{i}(t)$ and additive *r*(*t*) forms:

(3) $$Y(t)=\sum_{i=1}^{d}h_{i}(t)+r(t)\;$$

In the above formula, *d* represents the number of multivariate IMF layers decomposed *h*(*t*), *r*(*t*) is the *n* + 1 group signal IMF component, and *n* + 1 margins respectively correspond to the *n* + 1 element signal. Finally, the IMFs corresponding to the *n* high-frequency harmonic auxiliary channels are deleted from the (*n* + 1) element IMFs, and the IMFs of the target signal are reserved.

## 3. Transfer Entropy

Transfer entropy (TE) can effectively measure the potential directional transfer of information between two dynamic time series [43]. Given two stationary Markov processes *x* and *y*, the Markov process indicates that the random sequence of *x* and *y* occurs at a certain time and is only affected by the past finite time of the sequence. Then, the k-order Markov sequence defining *x* and *y* is:

(4) $$\begin{array}{l} P\left(x_{i}\left(1\right)|x_{i}{}^{\left(k\right)}\right)=P(x_{i}\left(n+1\right)|x_{i}\left(n\right),x_{i}\left(n-1\right),\\ \ldots ,x_{i}\left(n-k+1\right) \end{array}$$

(5) $$\begin{array}{l} P\left(y_{i}\left(1\right)|y_{i}{}^{\left(k\right)}\right)=P(y_{i}\left(n+1\right)|y_{i}\left(n\right),y_{i}\left(n-1\right),\\ \ldots ,y_{i}\left(n-k+1\right) \end{array}$$

According to the historical dynamic cross-correlation of the two time-series *x* and *y*, the transfer entropy $T_{y\to x}$ [34] can be defined as:

(6) $$\begin{array}{l} T_{y\to x}\left[x\left(1\right)|x^{\left(k\right)},y^{\left(l\right)}\right]=∭p\left[x\left(1\right)|x^{\left(k\right)},y^{\left(l\right)}\right]\\ \log_{2}\left[\frac{p\left(x\left(1\right)|x^{\left(k\right)},y^{\left(l\right)}\right)}{x\left(1\right)|x^{\left(k\right)}}\right]dx\left(1\right)dx^{\left(k\right)}dx^{\left(l\right)} \end{array}$$

where *k* and *l* are Markov process orders. Assuming that both time series are first-order Markov processes, then *k* = *l* = 1. The transfer entropy quantification describes the effect of the observed time series y on the future occurrence of another time series x. Introducing the delay parameter into Equation (6), it can be rewritten as the delay transfer entropy form [44,45]:

(7) $$\begin{array}{l} T_{y\to x}\left[x\left(1\right)|x^{\left(k\right)},y^{\left(l\right)}\left(\tau \right)\right]=∭p\left[x\left(1\right)|x^{\left(k\right)},y^{\left(l\right)}\left(\tau \right)\right]\\ \log_{2}\left[\frac{p\left(x\left(1\right)|x^{\left(k\right)},y^{\left(l\right)}\left(\tau \right)\right)}{x\left(1\right)|x^{\left(k\right)}}\right]dx\left(1\right)dx^{\left(k\right)}dy^{\left(l\right)}\left(\tau \right) \end{array}$$

## 4. Early Degradation State Recognition Method of Rotating Machinery Based on HA-MEMD and Transfer Entropy

**Figure 2:**
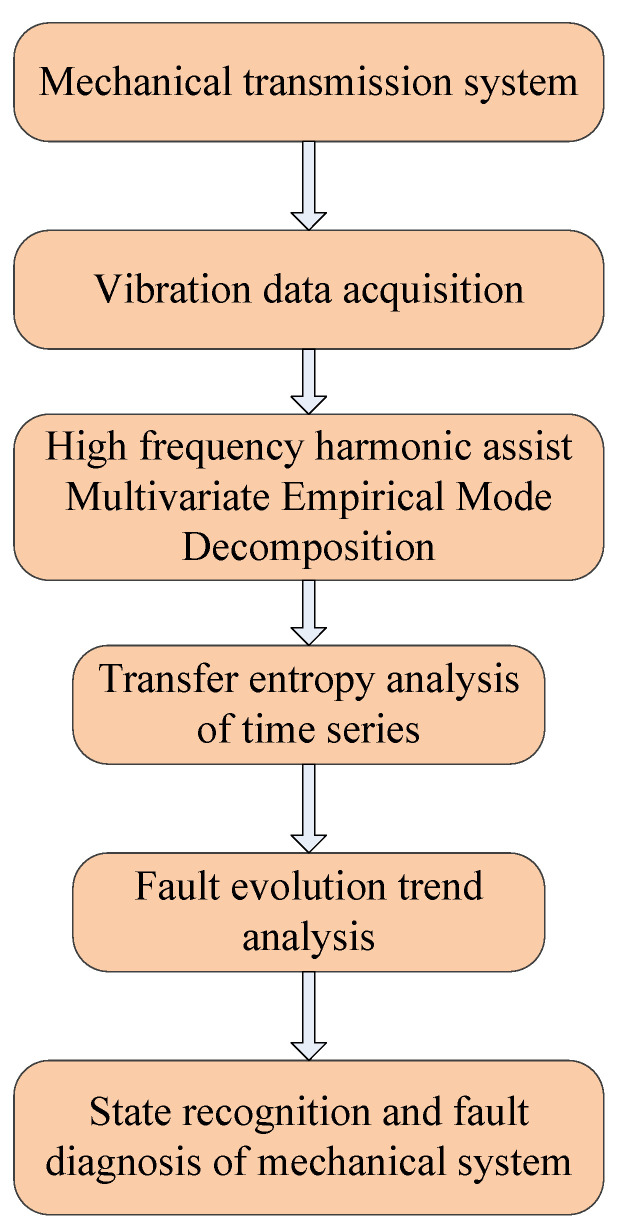
Mechanical early degradation state identification process.

The early failure signals of rotating machinery have the characteristics of weak features and low signal-to-noise ratios. HA-MEMD can effectively improve signal decomposition accuracy, achieve noise reduction of target signal, and improve signal to noise ratio. Transfer entropy can be used for the quantitative study of nonlinear synchronous coupling characteristics and information transfer between rotating machinery signals between different time–frequency scales, and it exhibits strong resistance to noise disturbance. This paper proposes an early fault detection method for rotating machinery based on the harmonic-assisted multivariate empirical mode decomposition method and transfer entropy, and establishes a rotating mechanical state evaluation index based on the HA-MEMD-TE to quantitatively describe the nonlinearity between historical state information under bearing fault conditions. To quantitatively describe the nonlinear coupling information and signal transmission characteristics between the historical state information under the condition of bearing fault., the following process is performed. Firstly, the signal is decomposed by HA-MEMD, the signal to be analyzed is denoised, the IMF component containing the main abnormal frequency characteristic information is selected for reconstruction, and the signal-to-noise ratio is highlighted to highlight the fault feature. Then, the transfer entropy algorithm, which is sensitive to the historical abrupt signal, is used. Passive entropy analysis is performed on the reconstructed signal to quantitatively describe the degree of nonlinear coupling between the signals to effectively evaluate and diagnose the operating state of the mechanical system. The principal process is as follows (As shown in Figure 2):

(1) Test data acquisition and denoising: The vibration signal is subjected to active noise cancellation by subjecting the acquired bearing vibration data to HA-MEMD decomposition, reconstructing the IMF components that contain key information, and removing the false components.
(2) Time series transfer entropy analysis: The transfer entropy between various time series is computed and the performance index for an accurate reflection of the bearing fault evolution trend is established.
(3) Mechanical fault evolution analysis and fault diagnosis: The stable and accurate fault thresholds are set based on early and stationary monitoring of transfer entropy variation, the transfer entropy trend is monitored in real time, and the rotating machinery is subjected to state recognition and fault diagnosis.

## 5. Numerical Simulation

To verify the filter characteristics and noise robustness of the HA-MEMD method, two given signals were chosen for a numerical simulation test, and a study was performed on the comparison between the proposed method and the results of EEMD and NA-MEMD.

### 5.1. Intermittent Signal

Modal aliasing may cause the IMF components obtained through EMD decomposition to contain different time scales. This leads to a chaotic time-frequency distribution, thereby making it difficult to identify the physical significance of each modal component and impairing the bearing vibration signal denoising effect and fault feature extraction.

**Figure 3:**
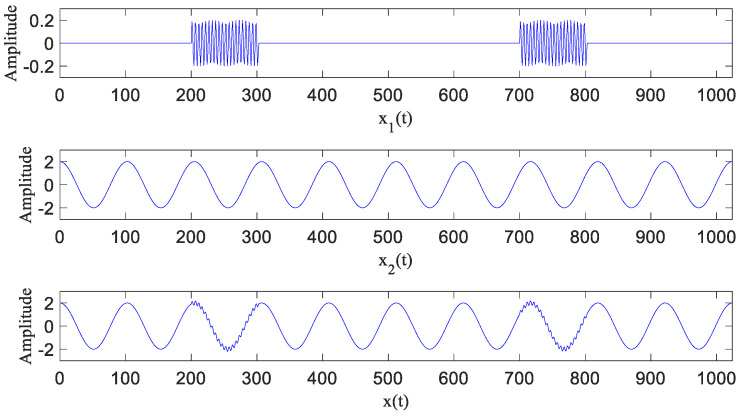
Intermittent simulation signal.

**Figure 4:**
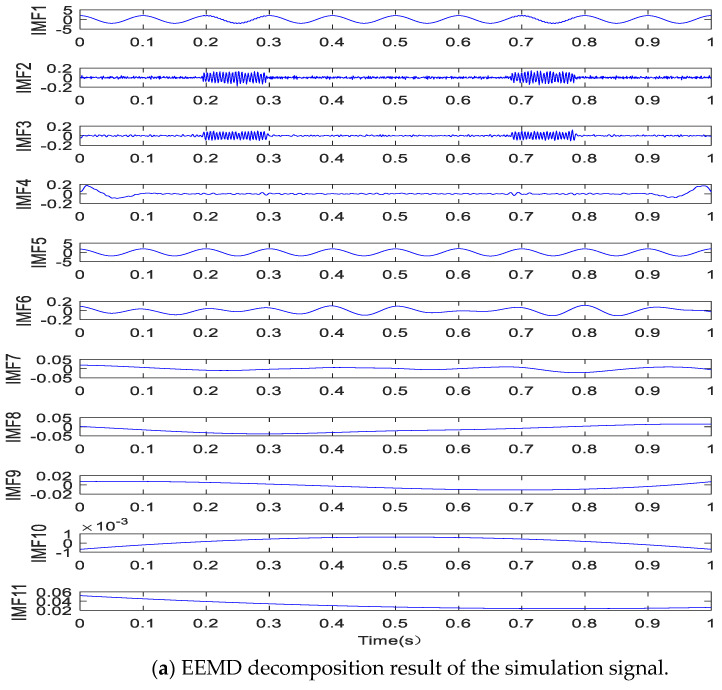

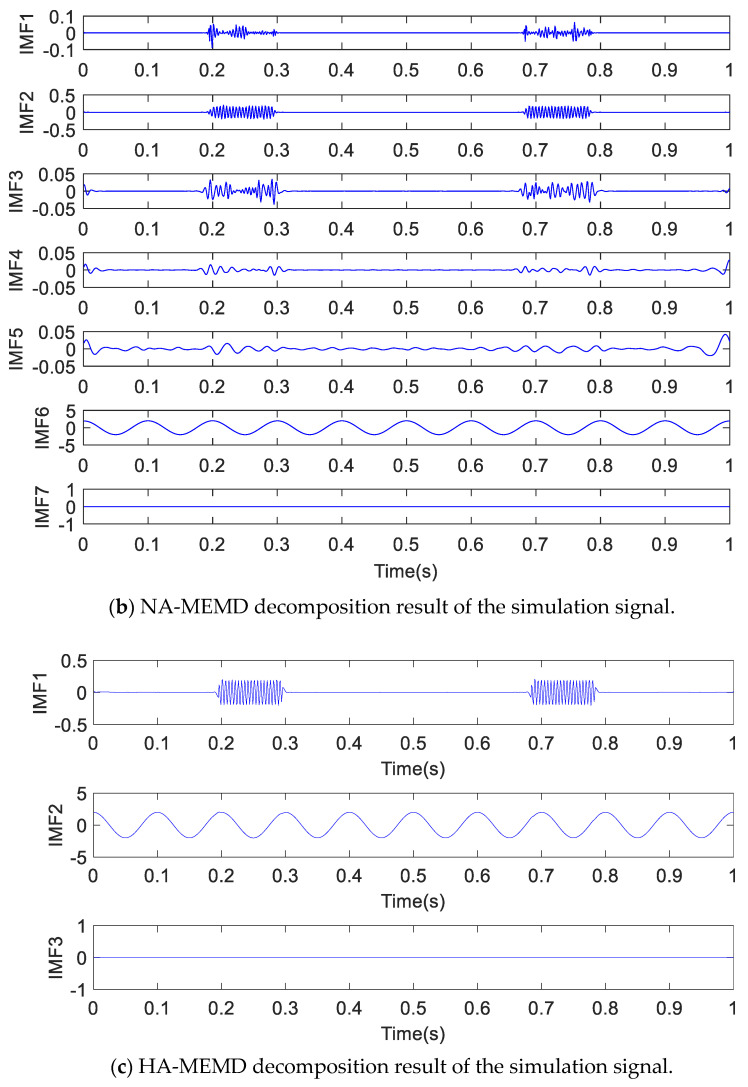
Comparative analysis of intermittent simulation signals.

To explain the effectiveness and superiority of HA-MEMD in the suppression of modal aliasing, the simulation signal *x*(*t*) shown in Figure 3—which is composed of a high-frequency intermittent signal *x*_1_(*t*) with an amplitude of 0.2 and a low-frequency cosine signal *x*_2_(*t*) with an amplitude of 1—was decomposed by EEMD, NA-MEMD, and HA-MEMD, respectively, and the results are shown in Figure 4. The frequency resolution of the simulation signal is 1 Hz.

The EEMD decomposition result of the numerical simulation is shown in Figure 4a. The simulation signal *x*(*t*) is decomposed into 11 MF components and a remainder term; the first 10 MF components are shown in the figure. IMF2 and IMF3 are associated with the high-frequency intermittent signal *x*_1_(*t*), while IMF5 is associated with the low-frequency cosine signal *x*_2_(*t*). The cross-correlation coefficients are 0.9067 between IMF2 and the interrupted signal *x*_1_(*t*), 0.9352 between IMF3 and *x*_1_(*t*), and 0.9992 between IMF5 and the low-frequency cosine signal *x*_2_(*t*). The decomposition brings about many noneffective IMF components (IMF6–IMF10). The figure indicates serious modal aliasing, where decomposition results in many false components.

NA-MEMD was used to decompose the simulation signal, and three white Gaussian noise channels with a variance of 0.2 were added. The decomposition results are shown in Figure 4b. The simulated signal is decomposed into five IMF components and a remainder term. Components *x*_1_(*t*) and *x*_2_(*t*) are clearly derived and respectively correspond to IMF2 and IMF6; the cross-correlation coefficient between IMF2 and the interrupted signal *x*_1_(*t*) is 0.9749. The decomposition results of HA-MEMD are shown in Figure 4c. The interrupted signal *x*_1_(*t*) and the low-frequency cosine signal *x*_2_(*t*) are clearly and accurately derived and correspond to IMF1 and IMF2, respectively; the cross-correlation coefficient is 0.9857 between IMF1 and the intermittent signal *x*_1_(*t*), and the cross-correlation coefficient is 1 between IMF2 and the low-frequency cosine signal *x*_2_(*t*). The decomposition result involves no modal aliasing.

According to the comparison result, the decomposition capacity of HA-MEMD is obviously superior to the other two methods; when compared with EEMD and NA-MEMD, HA-MEMD offers fewer iterations, as well as decomposition results that are more compliant with a practical signaling situation, and it has more definite physical significance. To summarize, the proposed method is superior to EEMD and NA-MEMD in terms of the suppression of modal aliasing and the improvement in decomposition accuracy, which confirms the effectiveness and superiority of the HA-MEMD method in the suppression of modal aliasing.

### 5.2. Rotating Machinery Early Fault Analog Signal

In order to test the capacity of the HA-MEMD decomposition shock modulation signal, the following simulation signal was designed and simulated:

(8) $$v(t)=x_{1}(t)\times x_{2}(t)\;$$

where *x*_1_(*t*) is a periodic exponential decay signal with a frequency of 18 Hz, the impact function per week is $2e^{-50}\sin(256\pi t)$, and the harmonic signal is $x_{2}(t)=\cos(1200\pi t)$. To verify the noise reduction capability of the HA-MEMD method, white noise with a signal-to-noise ratio of −5 dB was added to the original analog signal. The frequency resolution of the simulation signal is 1 Hz.

**Figure 5:**
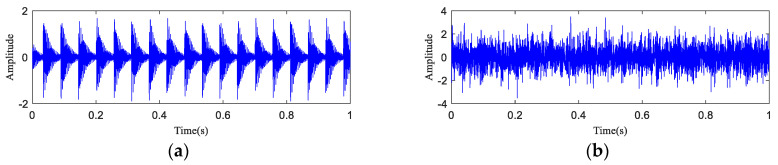

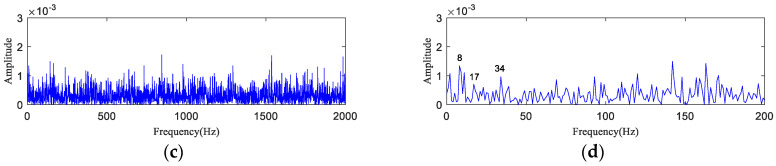
Simulation signal for rotating machinery failure. (**a**) Rotating machine fault simulation signal; (**b**) Time-domain waveform of the simulated signal containing noise (−5 dB); (**c**) Spectrogram of the simulated signal containing noise (−5 dB); (**d**) Partially magnified image containing the spectrum of the noise simulation signal (0–200 Hz).

**Figure 6:**
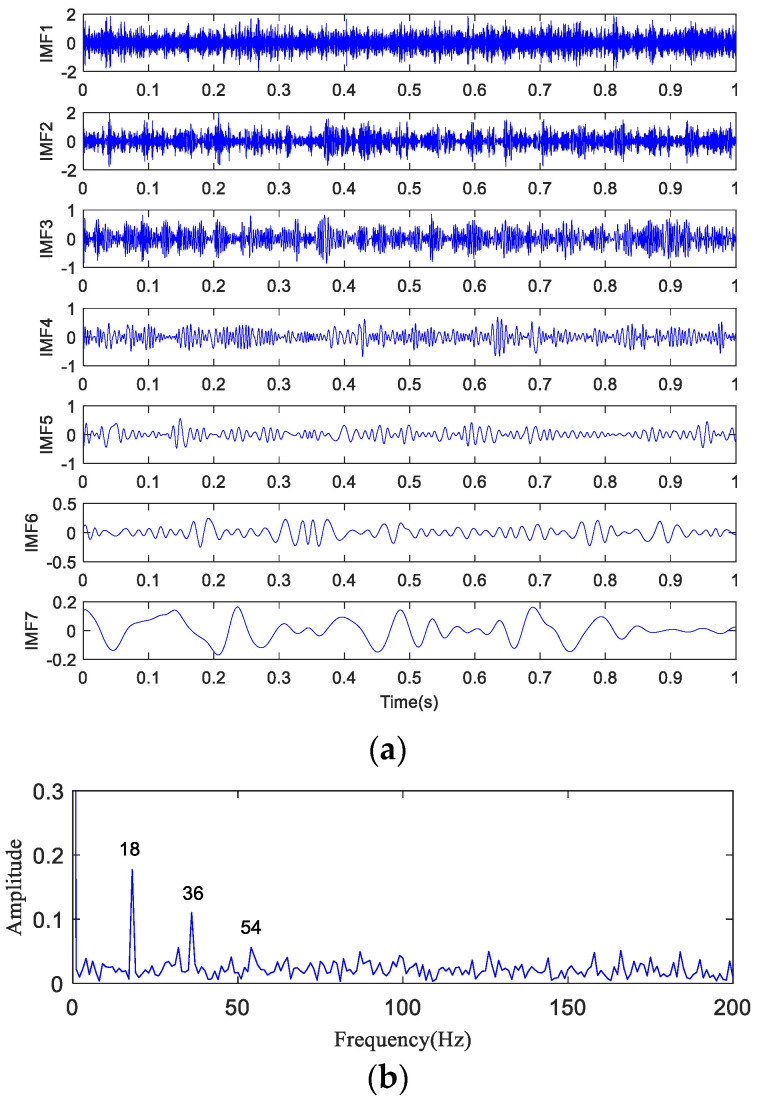
HA-MEMD analysis results of simulated signals. (**a**) HA-MEMD decomposition results of rotating machinery fault simulation signals; (**b**) Hilbert demodulation result of the simulated signal.

The time-domain waveform and spectrogram of the noisy simulation signal are shown in Figure 5a,b, according to which the 18 Hz impact characteristic frequency is drowned in noise. Only 8 Hz, 17 Hz, and 34 Hz unrelated frequencies can be seen in the spectrogram, which is difficult to reflect on a spectrogram. The HA-MEMD decomposition result of the simulation signal is shown in Figure 6a; Figure 6b shows the amplitude spectrum obtained through Hilbert demodulation after signal reconstruction of the first three IMF components that contain the main information of each channel. The 18 Hz shock signal and its doubling signal (36 Hz, 54 Hz) are accurately extracted, while the amplitude of the white noise component is small in the amplitude spectrum. The high-frequency harmonic-assisted multivariate empirical mode decomposition offers high local decomposition accuracy, and the result shows that HA-MEMD decomposition can effectively suppress a variety of harmonic signals and noise.

## 6. Experimental Research

### 6.1. Test Introduction

**Figure 7:**
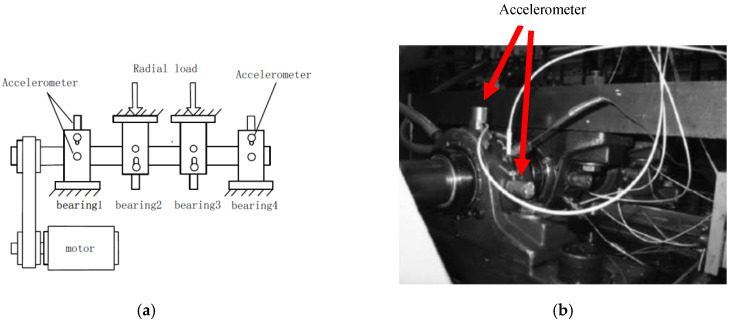
Rolling bearing Fault experimental platform. (**a**) Rolling bearing Fault experimental platform; (**b**) Sensor installation position.

**Figure 8:**
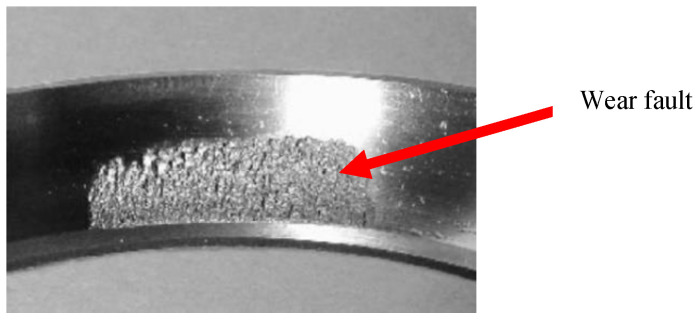
Bearing outer ring wear fault.

The test data were from the U.S. Intelligent Maintenance Systems (IMS) [46]. On the test stand shown in Figure 7a, the four bearings to be tested were respectively installed on the shaft, and a 6000-pound radial load was applied to the shaft and bearing. Two high-sensitivity integrated circuits piezoelectric (ICP) accelerometers were mounted on each bearing block to acquire the acceleration signals in X and Y directions; the sensor layout is shown in Figure 7b. The test conditions are shown in Table 1. After the whole test, the tester demolished the outer ring of the bearing and found that there was an obvious outer ring fault (Figure 8).

### 6.2. The Effect of Time Series Length on the Calculation of Transfer Entropy

When it comes to large-sized mechanical equipment, accidental failures bring about huge financial losses. To alleviate the risk of sudden failure arising from an early fault of rotating machinery, fault feature extraction and diagnostic methods are required that accurately describe the fault status, require a short length of data, etc. For the analysis and processing of a practical rotating machinery fault signal, signal length is an important factor affecting real-time analysis; the fast and timely analysis of a mechanical fault signal offers a strong guarantee for the establishment of a maintenance policy.

**Figure 9:**
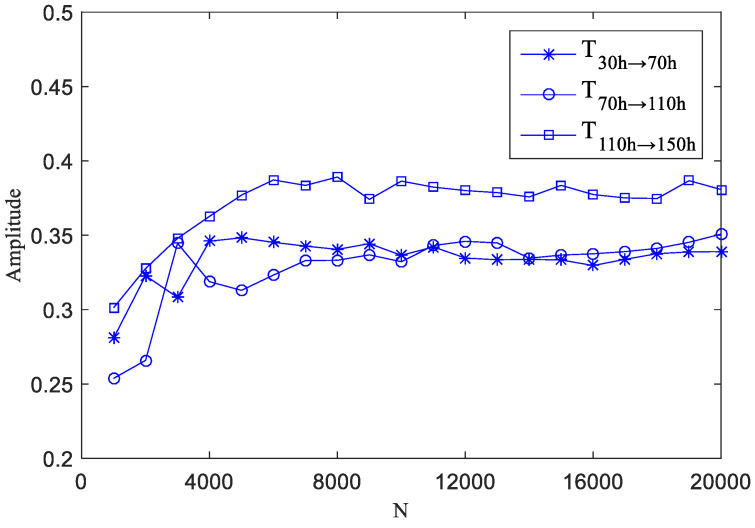
The transfer entropy of the length of different time series.

To study the effect of signal length on phase transfer entropy, the authors performed an analysis based on test data acquired at different time points of the bearing fault test stand operation. The transfer entropy of the operation was computed for the following: 30 h → 70 h (T30 h → 70 h), 70 h → 110 h (T70 h → 110 h), and 110 h → 150 h (T110 h → 150 h). Then, 1000, 2000, 3000, …, 20,000 data points were taken respectively for the three sets of data. The variation of the phase transfer entropy value with the signal length was determined through simulation; the time series length-specific transfer entropy is shown in Figure 9.

As shown in Figure 9, when the data length is less than 6000, the transfer entropy fluctuates significantly, but it tends to be stable when the data length is more than 6000. Considering the stability of the computation result and the computation cost, this paper sets the data length at 10,240.

### 6.3. Early Fault Detection of Rotating Machinery Based on the HAMMED-TE Method

**Figure 10:**
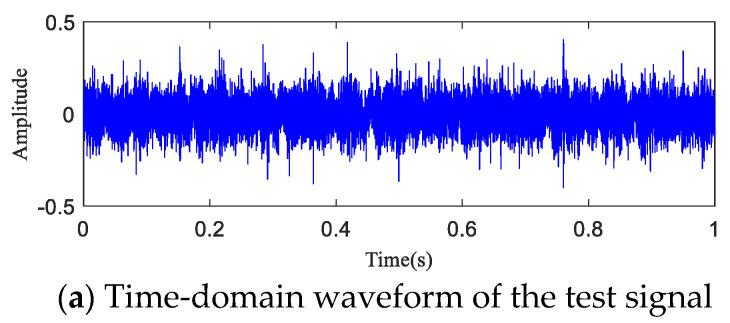

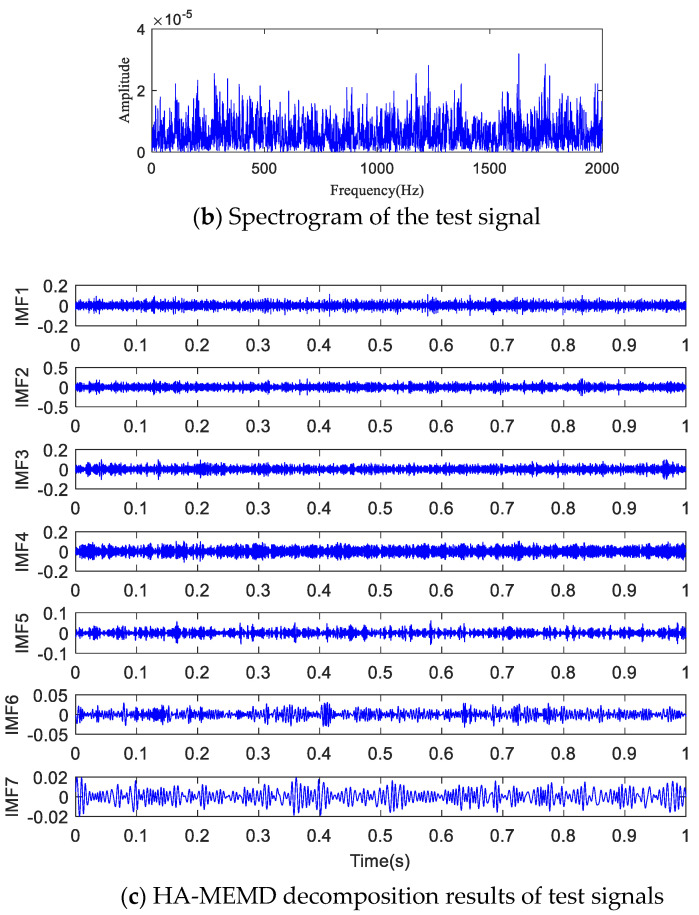
Analysis result the test signal (t = 30 h).

**Figure 11:**
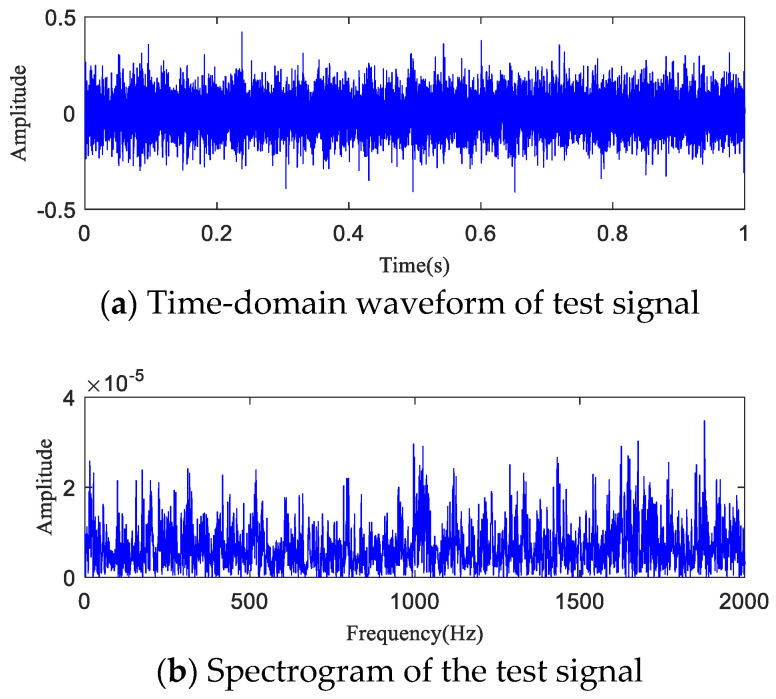

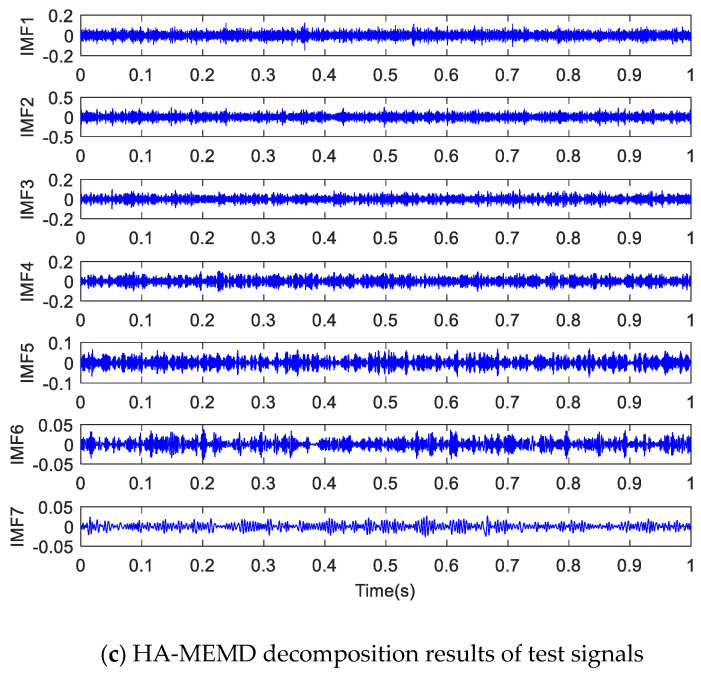
Analysis result test signal (t = 90 h).

**Figure 12:**
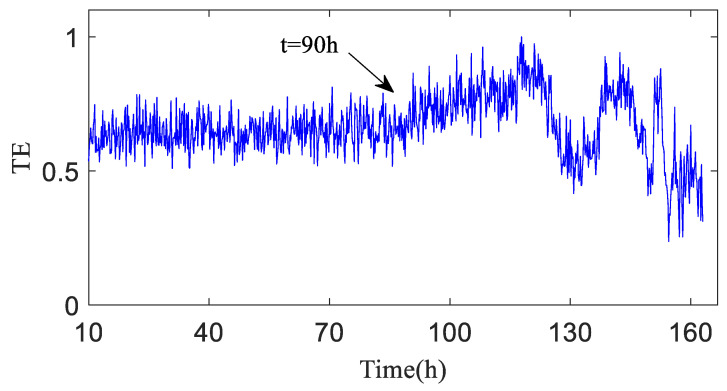
Transfer entropy of unknown operation data → failure-free data $T_{X\to Y}$ (λ = 1).

The test data (h30 and h90) the time-domain plots, frequency-domain plots, and HA-MEMD decomposition results of the operation data acquired at both time points are given in Figure 10 and Figure 11. are shown in Figure 10 and Figure 11. HAMEMD-TE was used for the analysis of the test data. First, any sample that was taken approx. 30 h after the stable operation of the test was randomly chosen as the bearing health data, which was set to *Xi*(*t*) (“*i*" was obtained randomly). Unknown operation data samples taken thereafter were set to *Yn*(*t*) (*n* = *i* + 1, *i* + 2, …,984 − *i*). *Xi*(*t*) and *Yn*(*t*) were subjected to to HA-MEMD adaptive decomposition, respectively. As shown in Figure 10c and Figure 11c, the fourth component of the decomposition result only contains a little time-domain information, so the top three IMF components containing the main fault information were subjected to summation and reconstruction. Finally, the signal reconstruction for both statuses was subjected to transfer entropy calculation to the nonlinear coupling information and signal transfer characteristics between the status information of the bearing fault being subjected to quantitative description, as follows: and the transfer entropy of failure-free status data → unknown operation status data $T_{X\to Y}$ (Figure 12). and the transfer entropy of unknown operation status data → failure-free status data $T_{Y\to X}$ (Figure 12) being calculated.

As shown in Figure 12, $T_{X\to Y}$ is totally dependent on historical data, and the entropy is relatively stable before h90, which means no early fault has developed in the bearing. The $T_{X\to Y}$ value rises progressively after h90, indicating that the variation of the bearing operation status is increasingly correlated with its own status, and that the bearing enters the early fault state from the failure-free state when the transfer entropy changes greatly. After 120 h of bearing operation, the $T_{X\to Y}$ transfer entropy fluctuates obviously, which indicates the mid-to-late stage of the bearing fault; that is to say, the fault is serious.

**Figure 13:**
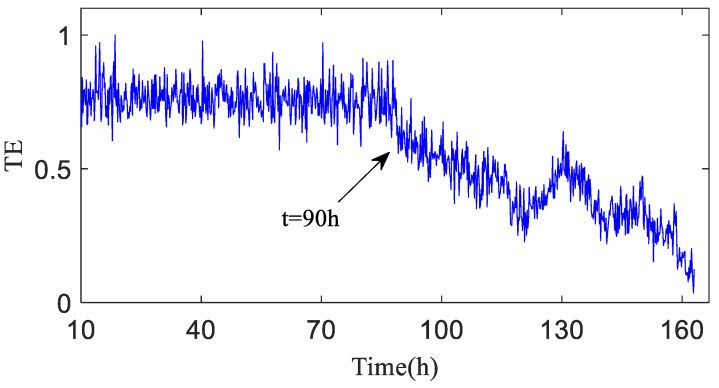
Transfer entropy of failure-free data → unknown operation data $T_{Y\to X}$.

Figure 13 shows the transfer entropy $T_{Y\to X}$ of unknown operation data → failure-free data. As shown in the figure, the variation trend of the transfer entropy $T_{Y\to X}$ accurately characterizes the entire operation status of the bearing. It is observed that no fault develops in the bearing before h90 when the transfer entropy is high and stable, which indicates that the unknown operation data of the bearing is significantly affected by previous failure-free data, and that the coupling is strong between time series. After 90 h of operation, bearing 1 develops an early fault in the outer race, and the vibration signal randomness changes, while the dynamic behavior changes suddenly. As shown in Figure 13, $T_{Y\to X}$ tends to decrease, which demonstrates that the effect of the previous failure-free data on the unknown operation data of the bearing decreases progressively, and that the degree of nonlinear coupling between time series decreases; after 120 h of the bearing operation, $T_{Y\to X}$ drops sharply.

**Figure 14:**
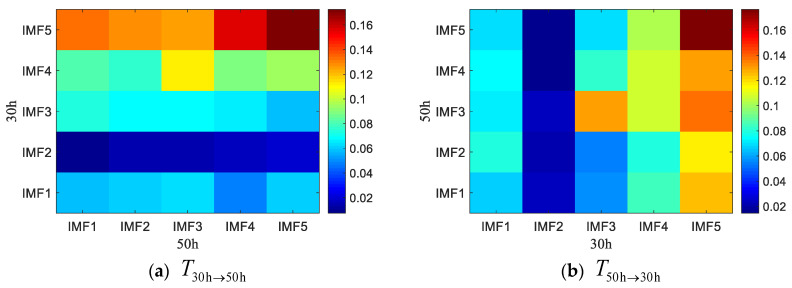
HAMEMD-TE values in different directions and different frequency bands during the 30th and 50th hours of the test bed operation.

**Figure 15:**
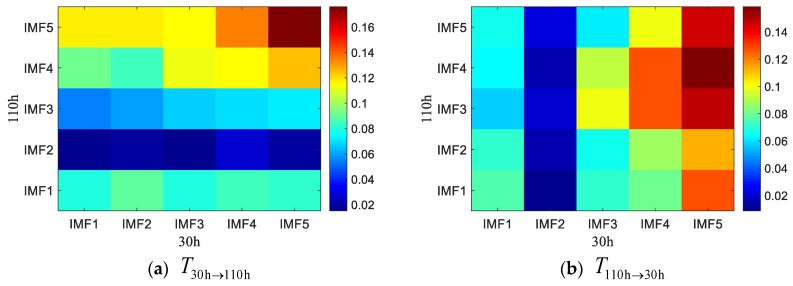
HAMEMD-TE values in different directions and different frequency bands during the 30th and 110th hours of the test bed operation.

For further study on the coupling characteristics of the rotating machinery fault signal between different frequency bands at different time points in different coupling directions, the HA-MEMD-TE for two different statuses at the time points h30 (failure-free state), h50 (failure-free state), and h110 (fault state) of operation of the rolling bearing test stand were chosen for analysis. First, HA-MEMD adaptive decomposition was performed to get several IMF components from high to low frequencies. Then, the transfer entropy values $T_{30h\to 50h}$ and $T_{50\to 30}$, corresponding to the IMF for the data acquired at h30 and h50, and the transfer entropy values $T_{30h\to 110h}$ and $T_{110\to 30}$, corresponding to the IMF for the data acquired at h30 and h110, were calculated. The results are given in Figure 14 and Figure 15. According to the comparison, as shown in Figure 14a, the strength of the coupling from components IMF1–IMF5 obtained through HA-MEMD decomposition of the data acquired at h30 of the test stand operation to the high-frequency component IMF1 obtained at h110 is better than before. In $T_{110h\to 30h}$, the strength of the coupling from IMF1–IMF5 to IMF4–IMF5 is high.

It can be observed from the analysis above that the entire evolution process (from nothing through minor to obvious) of an early weak fault in a rolling bearing is accurately characterized by the following tendency of the transfer entropy: $T_{X\to Y}$ of failure-free data → unknown operation data and the transfer entropy $T_{Y\to X}$ of unknown operation data → failure-free data. Compared with time-domain statistics indicator, the HA-MEMD-TE technique detects an early weak fault more than 20 h in advance. It can more accurately reflect the real running status of the rolling bearing, which demonstrates the effectiveness of HA-MEMD-TE in the testing of an early weak fault in a rolling bearing, having provided a reliable basis for mechanical system status monitoring and evaluation.

### 6.4. Research on Noise Robustness of HAMEMD-TE Method

**Figure 16:**
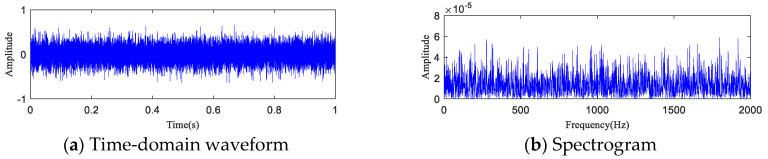
t = 90 h Experimental data (−5 dB).

**Figure 17:**
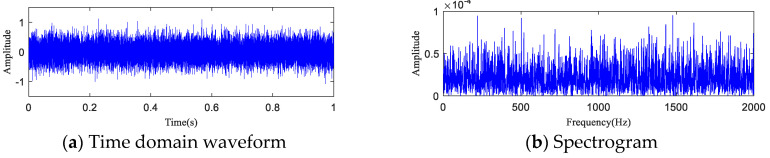
t = 90 h Experimental data (−10 dB).

**Figure 18:**
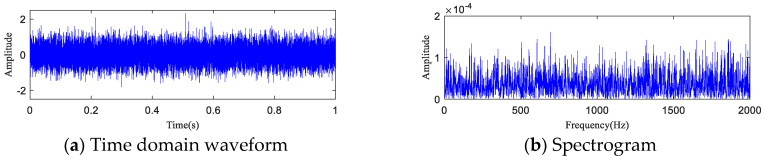
t = 90 h Experimental data (−15 dB).

**Figure 19:**
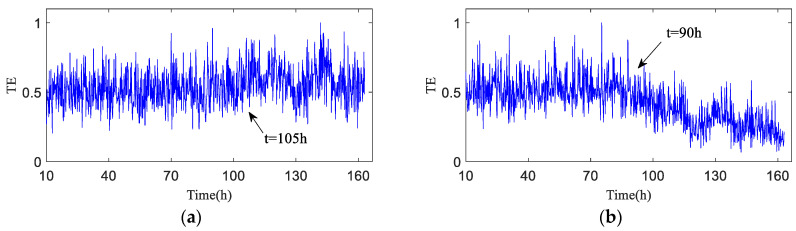
Test data based on HA-MEMD-TE analysis results (−5 dB). (**a**) Transfer entropy of unknown operation data → failure-free data $T_{X\to Y}$; (**b**) Transfer entropy of failure-free data → unknown operation data $T_{Y\to X}$.

**Figure 20:**
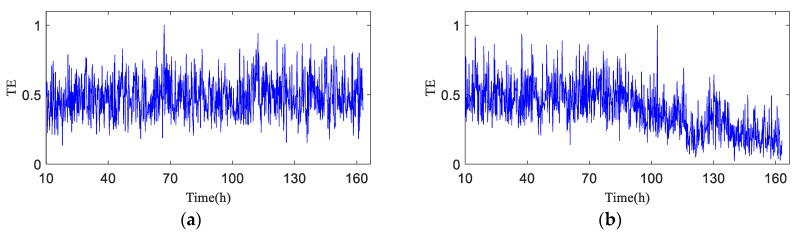
Test data based on HA-MEMD-TE analysis results (−10 dB). (**a**) Transfer entropy of unknown operation data → failure-free data $T_{X\to Y}$; (**b**) Transfer entropy of failure-free data → unknown operation data $T_{Y\to X}$.

**Figure 21:**
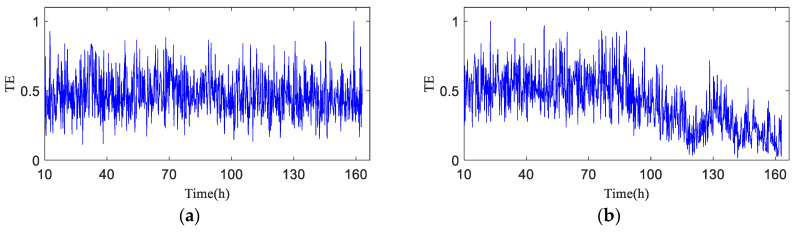
Test data based on HA-MEMD-TE analysis results (−15 dB). (**a**) Transfer entropy of unknown operation data → failure-free data $T_{X\to Y}$; (**b**) Transfer entropy of failure-free data → unknown operation data $T_{Y\to X}$.

For further quantitative study on the noise robustness of the HA-MEMD-TE technique, strong noise with SNRs of −5 dB, −10 dB, and −15 dB was additionally added to all data, respectively, based on the background noise and the complex excitation of the mechanical transmission system that disturb the test data described in the previous section. Figure 16, Figure 17 and Figure 18 respectively show the time-domain plot and spectrum containing the noise signal at a certain time point. The noisy data were analyzed using the HA-MEMD-TE technique. The analysis results are shown in Figure 19, Figure 20 and Figure 21, where it is observed that, despite the disturbance by strong background noise, transfer entropy can satisfactorily distinguish various stages of fault generation and evolution, and can accurately reflect the degree of nonlinear correlation between different running statuses of the bearing. From this point of view, HA-MEMD-TE remains effective for the detection of an early fault. As shown in Figure 20, when disturbed by a strong noise of −10 dB, the transfer entropy fluctuates significantly when the bearing runs for approx. 95 h, thereby accurately detecting the fault. This confirms the strong sensitivity and noise robustness to an early fault of the rotating machinery. Strong background noise and the complex excitation of the mechanical system cause a reduction in the sensitivity of transfer entropy in the fault feature estimation capacity, which demonstrates the necessity of denoising the data through HA-MEMD.

## 7. Summary and Discussion

In this paper, a new nonlinear signal processing method—high-frequency harmonic-assisted multi-empirical mode decomposition—is proposed to solve the problem of mode mixing in MEMD and NA-MEMD. The decomposition results show that the IMF has a higher decomposition accuracy, and the processing speed is more than 30% higher than that of NA-MEMD. Monitoring the variation in the additional energy loss during the power transfer of mechanical equipment is a valuable approach for equipment fault state recognition. The high-frequent harmonic-assisted multivariate empirical mode decomposition-transfer entropy method proposed in this paper can (1) describe the dynamic characteristics of energy coupling and information transmission directivity for the interaction of rotating machinery fault signal between different time–frequency scales, (2) reflect the multi-time–frequency scale function coupling characteristics of the rotating machinery fault signal, and (3) enable the quantitation and localization of a fault. Thus, the method provides a basis for mechanical fault evolution status recognition and fault diagnosis. A simulation signal and rolling bearing test signal were used to demonstrate the superiority of HA-MEMD in modal alias suppression and denoising capacity. The HA-MEMD decomposition of the test data and the appropriate IMF fusion effectively removed the substantive noise, helping to maintain the status characteristic components. For further quantitative study on the noise robustness of the HA-MEMD-TE technique, strong noise with SNRs of −5 dB, −10 dB, and −15 dB was additionally added to all data, respectively, based on the background noise and the complex excitation of the mechanical transmission system that disturb the test data described in the previous section. The test results show that, despite the disturbance by strong background noise, transfer entropy can satisfactorily distinguish various stages of fault generation and evolution, and can accurately reflect the degree of nonlinear correlation between different running statuses of bearing. The diagnosis of an early fault in rotating machinery is a subject that deserves in-depth study. The author’s next major research emphasis will be on HA-MEMD method improvement and its adaptation.

## Figures and Tables

**Table 1:** Full life test working condition of rolling bearing.

Rotating Shaft Speed	Sampling Frequency	Sampling Period	Sampling Time	Data Length	Sample Number
2000 r/min	20 kHz	10 min	1 s	20,480	984
